# HTLV-1 Tax targets centrosomal Cep63 protein

**DOI:** 10.1186/1742-4690-12-S1-P62

**Published:** 2015-08-28

**Authors:** Emilie Martin, Renaud Mahieux, Chloé Journo

**Affiliations:** 1 Équipe Oncogenèse Rétrovirale, Lyon, France; 2 Équipe Labellisée ≪ Ligue Nationale Contre le Cancer, Lyon, France; 3 Centre International de Recherche en Infectiologie, INSERM U1111-CNRS UMR5308, Lyon, France; 4 École Normale Supérieure de Lyon, Lyon, France; 5 Université Lyon 1, LabEx ECOFECT -Eco-evolutionary dynamics of infectious diseases, Lyon, France

## 

HTLV-1 Tax protein plays a key role in oncogenesis. Tax was previously shown to target centrosomal functions by interacting with several cellular proteins such as TAX1BP2 or RanBP1, ultimately leading to aneuploidy. However, it is still unclear how Tax disturbs centrosome organization. Using a yeast two-hybrid screen, we identified the centrosomal Cep63 protein, a regulator of centriole duplication and a key actor of the centrosome-dependent spindle assembly checkpoint, as a Tax-interacting protein. This finding is consistent with the findings of Simonis and colleagues. The interaction with endogenous as well as ectopically expressed Cep63 was confirmed by immunoprecipitation and Ni-NTA pulldown assays. We demonstrated that the N-terminal domain (amino acid 1 to 135) of Cep63 is both necessary and sufficient to interact with Tax. Interestingly, our immunofluorescence analyses show that the centrosomal distribution of endogenous Cep63 is significantly altered in U2OS cells expressing Centrin-GFP and Tax. The data presented here provide evidence that Tax alters centrosomal organization by targeting Cep63, a process that could lead to centriole amplification and defects in centrosome-dependent cell cycle checkpoint.

